# Extra-ampullary Peutz–Jeghers polyp causing duodenal intussusception leading to biliary obstruction: a case report

**DOI:** 10.1186/s13256-016-0990-8

**Published:** 2016-07-15

**Authors:** W. S. L. De Silva, A. A. Pathirana, B. D. Gamage, D. S. Manawasighe, B. Jayasundara, U. Kiriwandeniya

**Affiliations:** 1 Post-Graduate Institute of Medicine, University of Colombo, Colombo, Sri Lanka; 2 Department of Surgery, Faculty of Medical Sciences, University of Sri Jayewardenepura, Colombo, Sri Lanka; 3 Department of Pathology, Faculty of Medical Sciences, University of Sri Jayewardenepura, Colombo, Sri Lanka

**Keywords:** Peutz–Jeghers polyp, Duodenal intussusception, Biliary obstruction, Small bowel obstruction, Case report

## Abstract

**Background:**

Duodenal Peutz–Jeghers polyp is a rare cause of duodenal or biliary obstruction. However, a sporadic Peutz–Jeghers polyp leading to simultaneous biliary and duodenal obstruction has not been reported.

**Case presentation:**

We report a case of a 25-year-old Sri Lankan woman presenting with features of recurrent upper small intestinal obstruction and biliary obstruction. She had clinical as well as biochemical evidence of intermittent biliary obstruction. Evidence of duodenal intussusception was found in a computed tomography enterogram and a duodenal polyp was noted as the lead point. Marked elongation and distortion of her lower common bile duct with intrahepatic duct dilatation was also noted and the ampulla was found to be on the left side of the midline pulled toward the intussusceptum. Open polypectomy and reduction of intussusception were done and she became fully asymptomatic following surgery. Histology of the resected specimen was reported as a typical “Peutz–Jeghers polyp”. As there was not enough evidence to diagnose Peutz–Jeghers syndrome this was considered to be a sporadic Peutz–Jeghers polyp.

**Conclusion:**

Rare benign causes such as a duodenal polyp should be considered and looked for in initial imaging, when the cause for concurrent biliary and intestinal obstruction is uncertain, particularly in young individuals.

## Background

Duodenal polyps are commonly found as an incidental finding during endoscopic evaluations of the upper gastrointestinal (GI) tract. The prevalence of duodenal polyposis in routine upper GI endoscopies is 1.5 % [1]. These polyps rarely obstruct the lumen of the duodenum or become the lead point of a duodenal intussusception. Literature on such lesions resulting in biliary obstruction together with intestinal obstruction is rare. Peutz–Jeghers polyps occur as sporadic lesions or as a part of Peutz–Jeghers syndrome (PJS). They account for 1.8 % of all duodenal polyps [1]. Diagnosis of these polyps is readily made as they show characteristic histological features. Here we report our management experience of a young woman with a sporadic Peutz–Jeghers polyp causing a duodenal intussusception with simultaneous duodenal and biliary obstruction.

## Case presentation

A 25-year-old Sri Lankan woman presented with several episodes of central abdominal pain, abdominal fullness, and non-projectile vomiting for 1-week duration. The vomitus was non-bilious, with undigested food particles and was noted particularly 1 to 2 hours after meals. Episodes of vomiting were accompanied with colicky central abdominal pain but these symptoms were only mild and between attacks she was completely asymptomatic. As she had had at least five similar episodes over the last 3 years, she sought medical advice. Most of those episodes were self-limiting, but she had a couple of hospital admissions during which she was managed conservatively. During one episode, she had noticed pruritus and darkening of urine as well, which again resolved spontaneously. She had undergone an upper GI endoscopy 1 year earlier which did not provide a positive finding. She had a past history of rectal polypectomy, at the age of 16 years, when she was investigated for painless per rectal bleeding, the histology of which was consistent with a tubular adenoma. Follow-up colonoscopies had not detected any further polyps.

On examination, she had a body mass index of 20.3 kg/m^2^. She had a few scratch marks on her trunk and upper limbs confirming pruritus. She was not icteric or febrile. An abdominal examination was unremarkable and her gall bladder was not palpable. Succussion splash was not elicited. There was no mucocutaneous pigmentation.

**Fig. 1:**
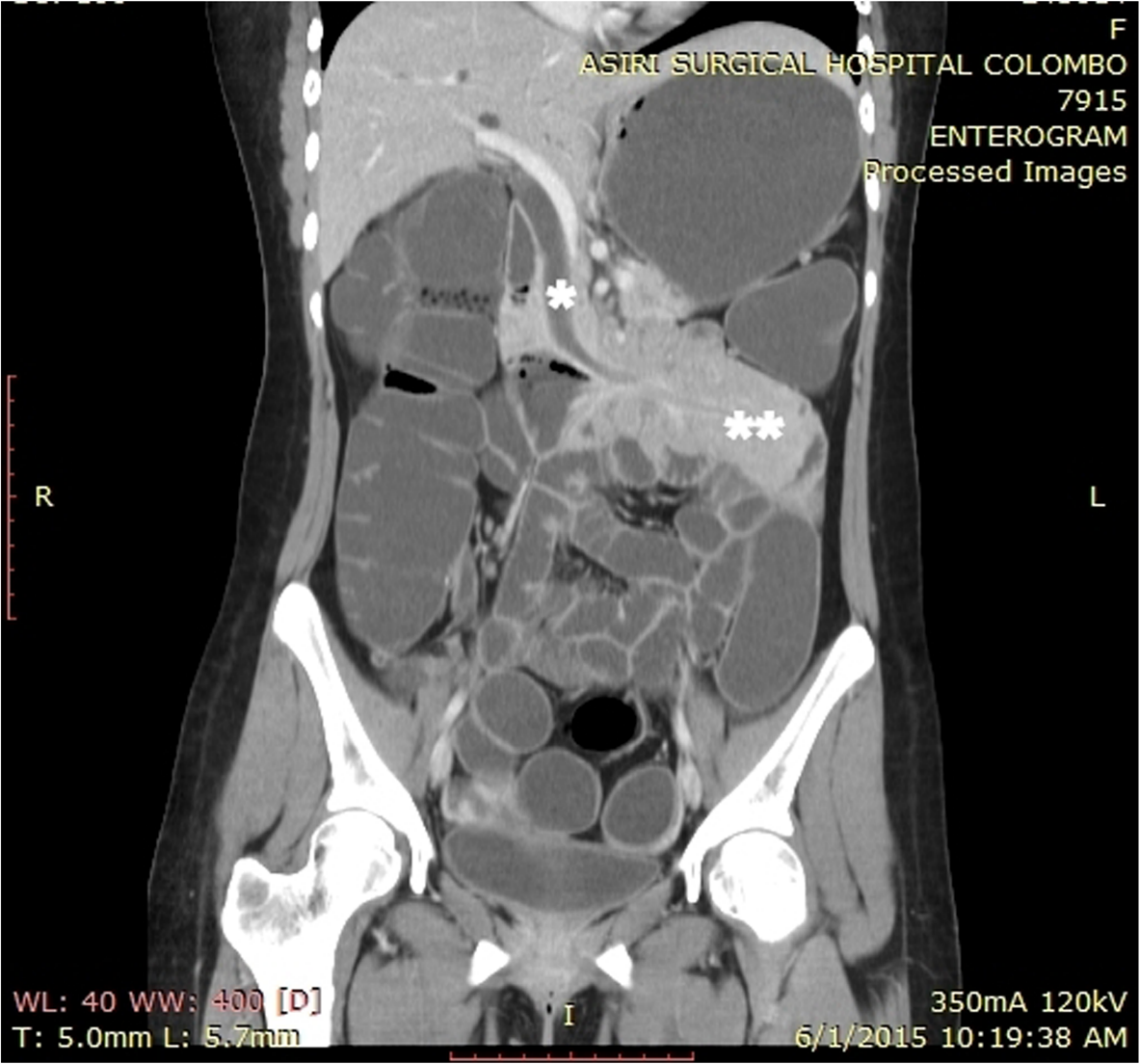
A coronal reconstruction of computed tomography enterogram showing elongated and dilated common bile duct with the lower end on the left side of the midline (*) and the intussusception with the intussusceptum in the proximal jejunum (**)

**Fig. 2:**
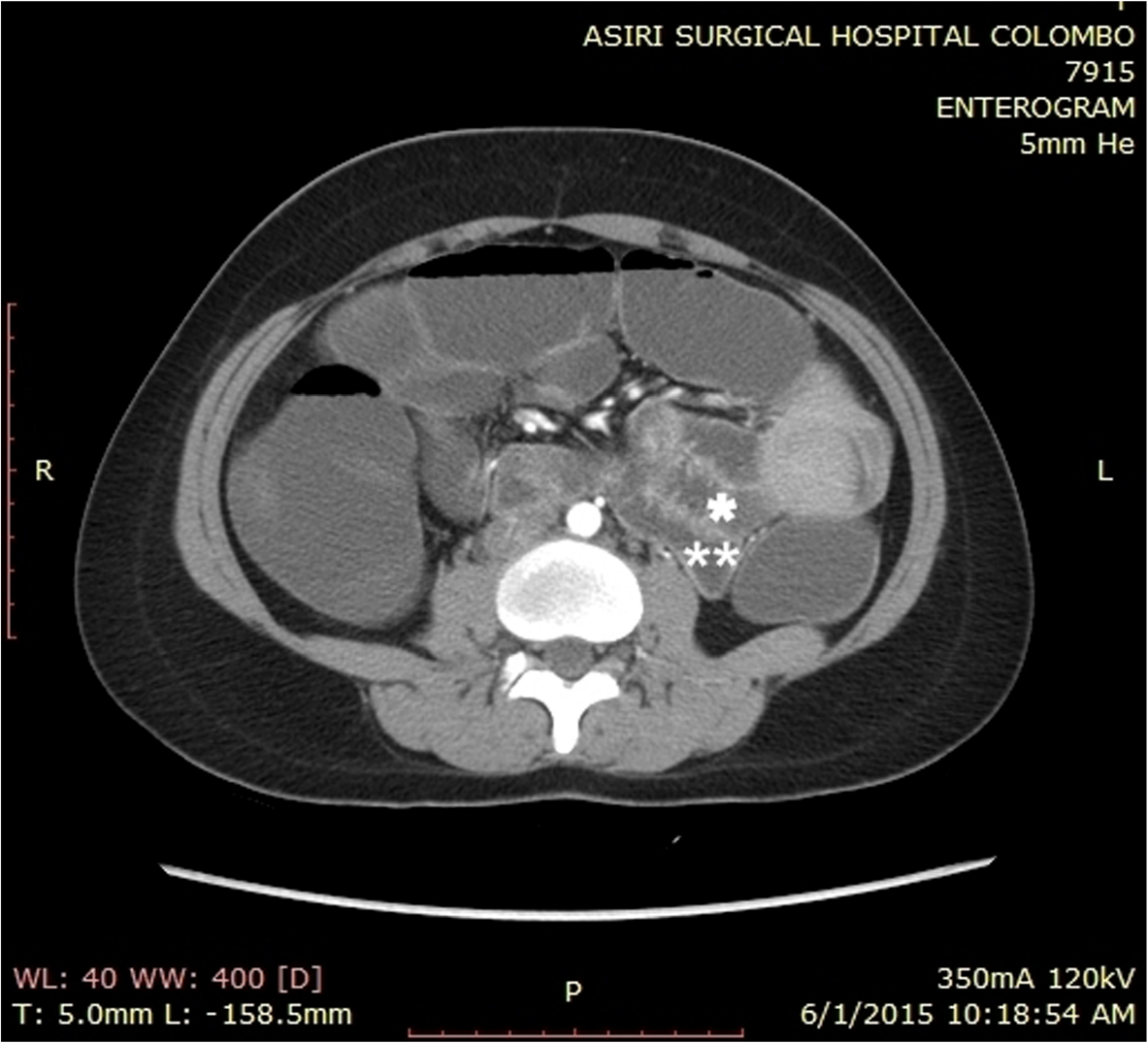
An axial section of the computed tomography enterogram showing the duodenal mucosal intussusceptum (*) and the intussusceptiens – the proximal jejunum (**)

**Fig. 3:**
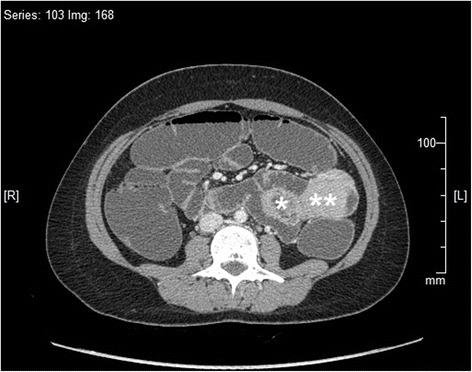
An axial section of the computed tomography enterogram showing the duodenal mucosal intussusceptum (*) and the duodenal polyp as the lead point of the intussusception (**)

There was biochemical evidence of biliary obstruction with alkaline phosphatase (ALP) of 896 IU/L, a total bilirubin of 2.6 mg/dl and direct bilirubin of 2.2 mg/dL. An ultrasound scan of her abdomen detected intrahepatic and extrahepatic duct dilatation with a dilated common bile duct (CBD) of 11.5 mm without evidence of gallstones or CBD stones. She was found positive for fecal occult blood and was subjected to upper GI endoscopy and colonoscopy both of which were negative. Plain radiographies of her chest and abdomen were unremarkable. She was further investigated with a computed tomography (CT) enterogram and a magnetic resonance cholangiopancreatogram (MRCP). The CT enterogram revealed a large soft tissue mass causing duodenal intussusception into her proximal jejunum. Her CBD was found to be stretched to the left of the midline resulting in its dilatation of up to 12 mm at the lower end. MRCP also confirmed the absence of gall stones and other filling defects in her CBD (Figs. 1, 2 and 3). Based on these findings she underwent a small bowel enteroscopy which demonstrated a large duodenal polyp, originating from the second part of her duodenum and intussuscepting into the proximal jejunum beyond the duodenojejunal junction. The major duodenal papilla appeared stretched and elongated along the long axis of her duodenum. The rest of the enteroscopy study was normal. A biopsy was not taken because of the presence of intussusception.

**Fig. 4:**
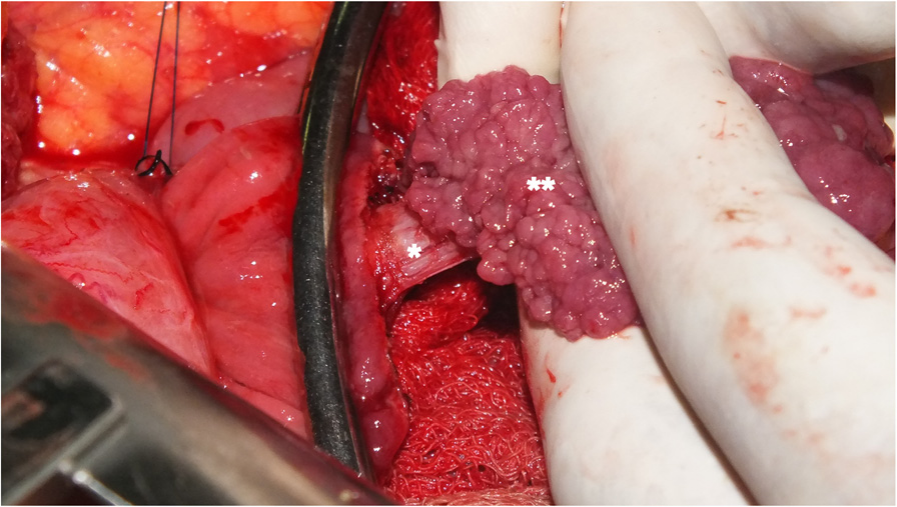
Intraoperative photograph showing the stalk of the polyp (*) and the main bulk of the polyp (**) after reduction of the intussusception

**Fig. 5:**
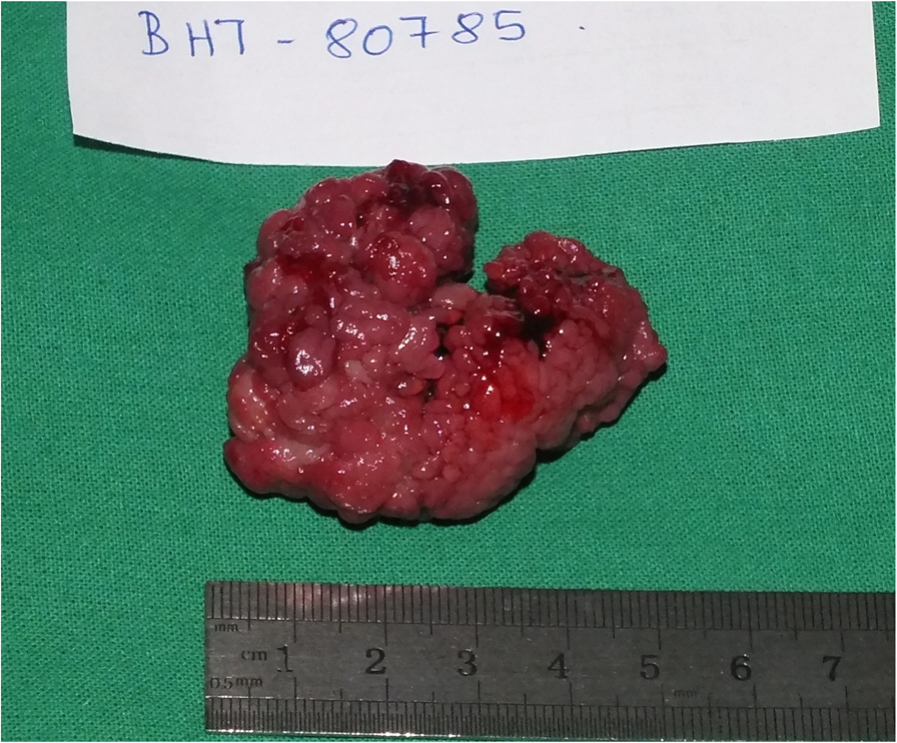
Macroscopic appearance of the polyp after resection. The stalk was retracted into the mass of polyp, thus not seen in the photograph

Even though it was possible to negotiate the scope beyond the polyp it was decided to go ahead with open surgery after two multidisciplinary team meetings because of the large size of the polyp, its critical location, and the presence of the intussusception. An intraoperative upper GI endoscopy was performed to localize the polyp because the polyp was not readily palpable through her duodenal wall. An oblique duodenotomy was done and a large polyp with a broad and long stalk was found. The origin of the stalk was at the second part of her duodenum, 1 cm below the ampulla. A polypectomy was done and the intussusception was reduced and the duodenum was closed transversely with 5/0 polydioxanone sutures (Figs. 4 and 5). She had an uncomplicated recovery and was discharged on sixth postoperative day.

**Fig. 6:**
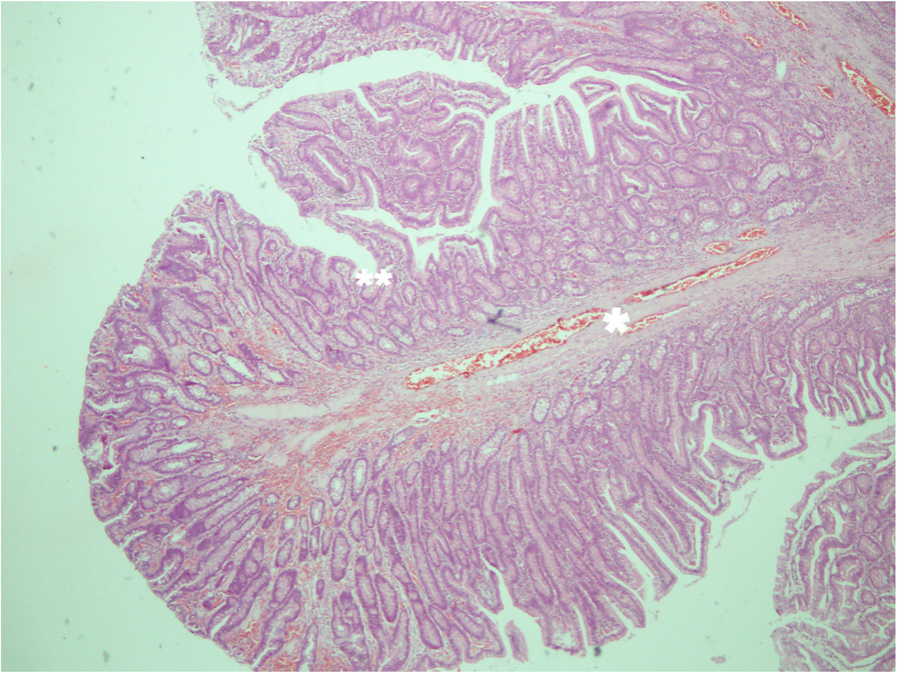
Microscopic architecture of the polyp showing villous structures containing a core of smooth muscle (*) and morphologically normal small intestinal mucosa overlying the villi (**)

**Fig. 7:**
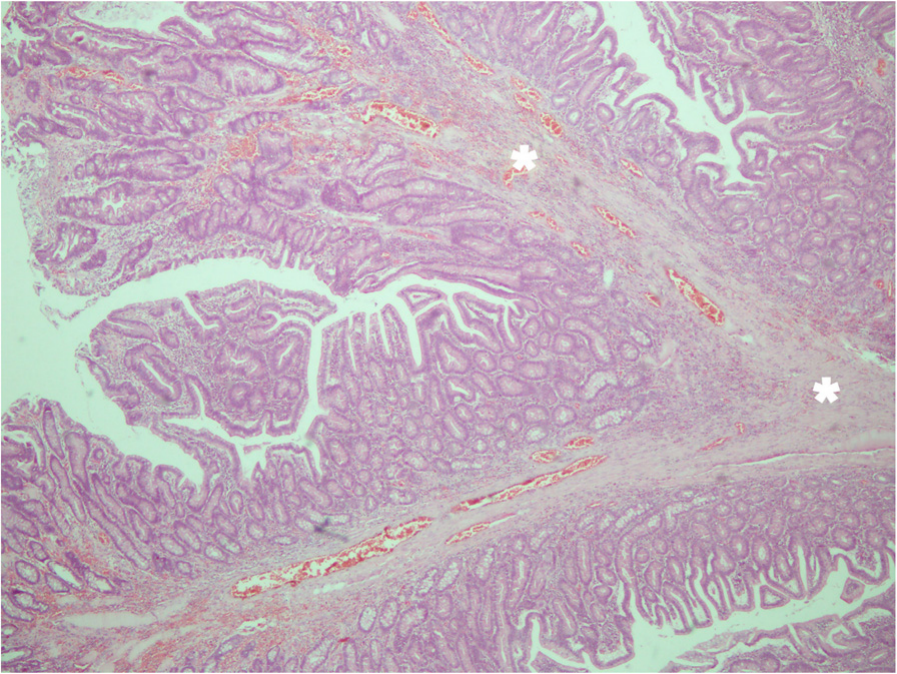
Microscopic architecture of the polyp showing branching points of villous structures containing a core of smooth muscle (*)

On macroscopic examination the specimen was a polyp of 50 × 45 × 30 mm in size with a broad, 20 mm long stalk (Fig. 5). On microscopic examination it was composed of a branching villous structure of small intestinal mucosa containing a core of smooth muscle. The overlapping mucosa was histologically normal. Thus, microscopically this was a hamartomatous polyp consistent with a Peutz–Jeghers polyp [2] (Figs. 6 and 7).

Our patient became completely asymptomatic following surgery. She is followed up in general surgical clinic and is scheduled for routine upper GI endoscopy surveillance every 3 years.

## Discussion

A non-ampullary duodenal polyp is a rare cause of biliary obstruction [3–6]. In several case reports juxta-ampullary polyps have caused both biliary and duodenal obstruction due to their large size [3–5]. Gentile *et al*. have reported a case of ampullary distortion following open duodenal polypectomy, as the culprit for biliary obstruction [6]. Duodenal intussusception due to Peutz–Jeghers polyposis has also been reported [3, 6]. However, to the best of our knowledge a case of a Peutz–Jeghers polyp causing duodenal intussusception resulting in both duodenal and biliary obstruction at the same time has not been reported. In our patient, the ampulla was distorted by the pull from the intussusceptum. The pull on the ampulla was so marked that the lower half of her CBD was found on the left side of the midline in a CT scan.

PJS is diagnosed if two out of the three diagnostic criteria (family history, mucocutaneous pigmentation, and intestinal hamartomatosis with typical Peutz–Jeghers-type histology) are fulfilled. In cases in which patients had a Peutz–Jeghers polyp that caused biliary obstruction, the patients had been diagnosed as having PJS [3–6]. Our patient did not have any of the other features for the diagnosis of PJS [7, 8]. *STK11* gene testing which can be done to confirm PJS in complement with previous criteria is not available in Sri Lanka [9, 10]. Thus, this lesion was considered a sporadic Peutz–Jeghers polyp.

An initial upper GI endoscopy of our patient did not detect any abnormality. This was probably because the lesion originated beyond the ampulla of Vater and the main bulk of the polyp was in the proximal jejunum as the lead point of the intussusception. Since upper GI endoscopies are commonly carried out only up to the second part of duodenum, evidence of polyps can be missed. This necessitated a CT enterogram to detect the exact abnormality.

Treatment options adopted previously for similar cases were open polypectomy [6], limited duodenectomy [3], Whipple procedure [4], and endoscopic resection [11]. Data on the safety and efficacy of endoscopic resection of duodenal polyps are sparse [11]. The size of successfully resected polyps from a reported case series is only 15.1 ± 5.1 mm [12]. Because our patient had an intussusception in addition to the polyp this option was not considered.

Patients with PJS have a risk of GI malignancy and so do patients with sporadic Peutz–Jeghers polyps. Guidelines are available for the surveillance of patients with PJS, but not for patients with sporadic polyps. Our patient is scheduled for follow-up upper GI endoscopy every 3 years according to the guidelines for PJS [10].

## Conclusions

New onset obstructive jaundice is not common in young individuals in the absence of gallstones. When such symptoms are associated with features of upper intestinal obstruction, early CT/MR imaging would help to localize a structural anomaly. As there are no specific guidelines available to manage sporadic foregut polyps each case requires tailor-made management.

## Abbreviations

ALP, alkaline phosphatase; CBD, common bile duct; CT, computed tomography; GI, gastrointestinal; MRCP, magnetic resonance cholangiopancreatogram; PJS, Peutz–Jeghers syndrome
